# The end of medicine

**DOI:** 10.1017/S1478951526103228

**Published:** 2026-07-21

**Authors:** Benjamin Frush

**Affiliations:** Palliative Care, Hospital Medicine, Duke University School of Medicine, Durham, NC, USA

“You really need to work on your efficiency.”

Amidst the flurry of advice provided during my first feedback session of intern year, this line stuck with me. Through 2 weeks of residency, I was struggling to keep my head above water as the deluge of clinical, logistical, and bureaucratic tasks swelled around me. My senior resident, Mark, was patient and direct in his counsel; it was clear that I needed a system to complete my work more quickly.

I recognize in hindsight that his message resonated for 2 primary reasons: it was true, and, more importantly, it was actionable. Yet it has also become clear in the 7 years since this exchange that the question of *why* efficiency was important, or to what end it should be oriented, never entered my mind.

Instead, dutiful intern that I was, I threw myself into action. I picked the brains of senior residents to learn how to format my patient list, reveling in every follow-up box checked and discharged surname struck through. I developed a “smart-phrase” shortcut for every problem that might populate my austere (yet accurate) progress notes. In the throes of my MICU rotation, I went so far as to create a template to standardize the weighty goals-of-care conversations I would recurrently undertake with patients:

(Patient name) states that (***) is most important to (patient name). (Patient name) states that (he/she/they) most fears (***). Additionally, (***). In light of this, the patient has conveyed the desire to be (Full Code/DNAR).

As an intern, I morphed into a note-writing, pager-wielding, discharge-facilitating machine. And with every nod of approval from my attending or “strong work” encomium from my senior resident, my tank brimmed. Importantly, while my drive for efficiency lacked a clear purpose, it found a distinct post hoc justification. The sentiment shared by my fellow millennial trainees that we were cheap labor and readily exploitable allowed me to interpret my efficiency as a mode of virtuous self-protection, resistance even. “The longer you stay, the longer you stay” morphed from a truism to a doctrine.

Yet doctrines invite heretics. One day near the end of intern year, I reclined in a chair in a drab workroom, scrolling on my phone as I counted the minutes until sign-out, when I could turn over my column of freshly-checked boxes to the long call intern. My senior resident, James, entered and asked how I was doing.

“Great,” I answered, “Notes done, patients discharged, just killing time.” He furrowed his brow and asked what I thought about the patient in room 19, Mr. Jones.

“Labs look good, and he’s back to room air. I have his discharge summary prepped for tomorrow,” I responded, expecting affirmation. Instead, I was met with a frown and silence.

“That’s good,” he replied tepidly. Before I could ask what I was missing, he continued,

“He seemed down on rounds this morning. I circled back to check in. Apparently the anniversary of his wife’s death is this week.” I nodded solemnly, unsure what to do with this information. Screen for suicidal ideation? Social work consult? No doubt sensing my bemusement, James, not typically the sentimental type, squeezed my shoulder gently and walked out of the room.

While James said little in this interaction, I found myself strangely convicted, smarting from a silent gut-punch. Through the remainder of the intern year, as I tried to understand what this exchange signified, I came to slowly discern the question latent in his response, which I had neglected to that point: “Efficient for what?”

This question, cogent as it was in June 2019, faces trainees and practitioners with a newfound urgency today. In a post-COVID context in which the self-protective impulse of trainees has heightened, efficiency seems the primary recourse to protect one’s time outside of the hospital (Rosenbaum 2024). For a generation of students who increasingly view medicine as a stepping stone to a career away from the bedside, efficiency mitigates the temporal encumbrance of clinical training (Elsevier 2023). In a throughput-crazed medical system in which AI technology promises speed and accuracy exceeding those of human clinicians, efficiency is reified as a good in itself, even as we ponder what role doctors now play (Kolata 2026).

Yet efficiency, by definition, cannot be its own end. To be perfectly efficient would finally mean no more work to be done. Efficiency is what Aristotle would term an “instrumental” good – it only finds meaning when pursued in service of a greater purpose, or end. Yet if medicine collapses into bare efficiency, so too collapses our ability to discern a clear end that governs when and how efficiency should be prioritized relative to other goods we seek as clinicians.

While I am grateful for the feedback I received from Mark early in the intern year, I wish his admonition has been coupled with a sense of what I should be working *toward*, which James tacitly embodied. The goodness of efficiency, I have learned through role models like James, can only be realized when coupled with a firm sense of what our patients need from us, what the late clinician and bioethicist Edmund Pellegrino termed the “right and good healing action taken in the interests of the particular patient” (Pellegrino 1979). Learning to understand this “right and good healing action” – whether this involves acute medical intervention, careful shared-decision making, or sometimes a listening ear or quiet presence – is ultimately learning to practice justice, of rendering each patient their due from us as clinicians, when time is scarce. But to render such justice requires a degree of attention, diligence, and patience that a focus on bare efficiency crowds out.

As palliative care practitioners within a medical system so thoroughly focused on efficiency, we are afforded an opportunity to demonstrate to the patients we care for and the trainees we teach what a healthier relationship to time looks like. Because while my palliative care checklist today may contain different “to dos” than did my intern year checklist, I am tempted by the same urge to finish work as quickly as possible, even when the time “saved” is not accorded to patients and families who warrant more of it. And while novel technologies like artificial intelligence may prove beneficial in palliative care workflow, these technologies cannot ultimately render *judgments* about how efficiency should be located within a hierarchy of other goods. They cannot dictate when it is appropriate to slow down or speed up; they cannot comprehend the wisdom and weight of pregnant silence; they cannot determine what a “right and good healing action” may look like for a patient who is dying. Such judgments require *persons* who can understand how efficiency might be situated within the broader context of what a given patient or family needs in a specific moment – including when to slow down.

We find ourselves in a cultural moment which increasingly augurs the “end” of medicine as we know it – in which the question “What are [doctors] really good for?” is posed in earnest rather than irony (Kolata 2026). To the degree that the good in medicine is conflated with bare efficiency, this question begs the prior one I was forced to grapple with as an intern – “efficient for what?”

Absent an answer to this latter question – one that subordinates the good of efficiency to the right and good healing acts for particular patients – we forget the “end” of the work we do as clinicians, including those of us in the palliative care context. And in so doing, we may unwittingly hasten our own.
